# Usual intake of one-carbon metabolism nutrients in a young adult population aged 19–30 years: a cross-sectional study

**DOI:** 10.1017/jns.2023.38

**Published:** 2023-04-24

**Authors:** Phachara Jindasereekul, Wachira Jirarattanarangsri, Julaluk Khemacheewakul, Noppol Leksawasdi, Parameth Thiennimitr, Siraphat Taesuwan

**Affiliations:** 1 Faculty of Agro-Industry, Chiang Mai University, 155 Moo 2, Mae Hia, Meuang, Chiang Mai 50100, Thailand; 2 Cluster of Innovative Food & Agro-Industry, Chiang Mai University, 155 Moo 2, Mae Hia, Meuang, Chiang Mai 50100, Thailand; 3 Cluster of Agro Bio-Circular-Green Industry, Chiang Mai University, 155 Moo 2, Mae Hia, Meuang, Chiang Mai 50100, Thailand; 4 Faculty of Medicine, Chiang Mai University, 110 Intawaroros Road, Si Phum, Meuang, Chiang Mai 50200, Thailand; 5 Research Center of Microbial Diversity and Sustainable Utilization, Chiang Mai University, 110 Intawaroros Road, Si Phum, Meuang, Chiang Mai 50200, Thailand

**Keywords:** Adequate intake, Adult, Asian, Betaine, Dietary reference intake, Estimated average requirement

## Abstract

One-carbon nutrients play an important role in epigenetic mechanisms and cellular methylation reactions. Inadequate intake of these nutrients is linked to metabolic perturbations, yet the current intake levels of these nutrients have rarely been studied in Asia. This cross-sectional study surveyed the usual dietary intake of one-carbon nutrients (folate, choline and vitamins B2, B6 and B12) among Thai university students aged 19–30 years (*n* 246). Socioeconomic background, health information, anthropometric data and 24-h dietary recall data were collected. The long-term usual intake was estimated using the multiple-source method. The average usual intake levels for men and women were (mean ± sd) 1⋅85 ± 0⋅95 and 2⋅42 ± 8⋅7 mg/d of vitamin B2, 1⋅96 ± 1⋅0 and 2⋅49 ± 8⋅7 mg/d of vitamin B6, 6⋅20 ± 9⋅5 and 6⋅28 ± 12 μg/d of vitamin B12, 195 ± 154 and 155 ± 101 μg dietary folate equivalent/d of folate, 418 ± 191 and 337 ± 164 mg/d of choline, respectively. Effect modification by sex was observed for vitamin B2 (*P*-interaction = 0⋅002) and choline (*P*-interaction = 0⋅02), where every 1 mg increase in vitamin B2 and 100 mg increase in choline intake were associated with a 2⋅07 (*P* = 0⋅01) and 0⋅81 kg/m^2^ (*P* = 0⋅04) lower BMI, respectively, in men. The study results suggest that Thai young adults meet the recommended levels for vitamins B2, B6 and B12. The majority of participants had inadequate folate intake and did not achieve recommended intake levels for choline. The study was approved by the Ethics Committee at the Faculty of Medicine, Chiang Mai University. This trial was registered at www.thaiclinicaltrials.gov (TCTR20210420007).

## Introduction

**Figure 1.**
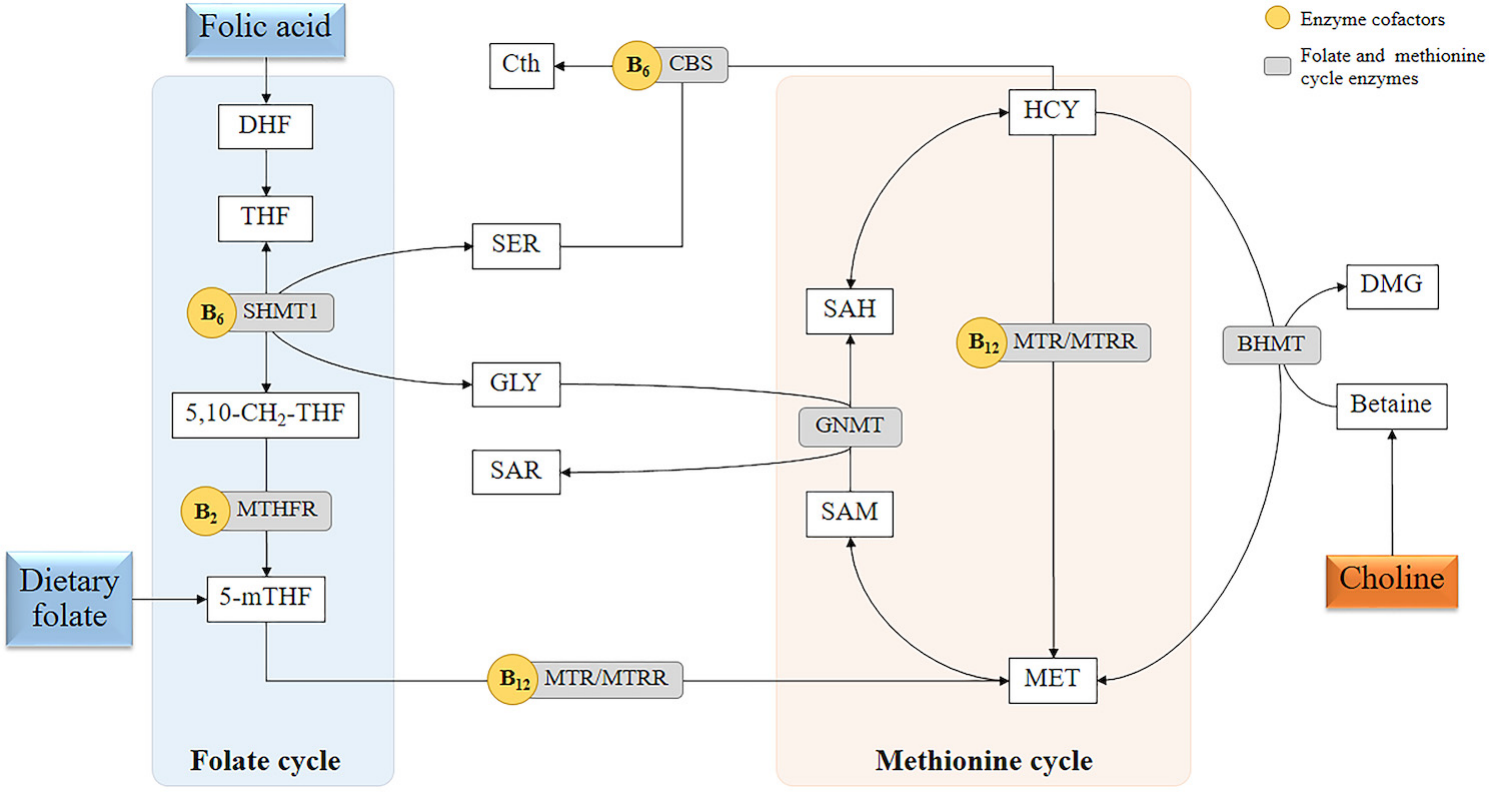
The roles of vitamins B2, B6 and B12, folate and choline in one-carbon metabolism. BHMT, betaine-homocysteine *S*-methyltransferase; CBS, cystathionine β-synthase; 5,10-CH_2_-THF, 5,10-methylene-THF; Cth, cystathionine; DHF, dihydrofolate; DMG, dimethylglycine; GLY, glycine; GNMT, glycine *N*-methyltransferase; HCY, homocysteine; MET, methionine; 5-mTHF, 5-methylTHF; MTHFR, 5,10-methylenetetrahydrofolate reductases; MTR, methionine synthase; MTRR, methionine synthase reductase; SAH, *S*-adenosylhomocysteine; SAM, *S*-adenosylmethionine; SAR, sarcosine; SER, serine; SHMT, serine hydroxymethyltransferase; THF, tetrahydrofolate.

One-carbon (1C) metabolism is a complex network of biochemical reactions that activates and transfers methyl groups. This network consists of folate, choline and methionine. The folate cycle produces nucleic acids that are used for DNA and RNA synthesis^(1,2)^, while choline is used for the synthesis of membrane phospholipids^(3)^. Both folate and choline intersect with the methionine cycle at the remethylation of homocysteine to methionine. Methionine synthase, a vitamin B12-dependent enzyme, catalyses the methyl group transfer from 5-methyltetrahydrofolate to homocysteine^(3)^. Homocysteine can also accept methyl groups from betaine, a choline derivative, in a reaction catalysed by betaine-homocysteine methyltransferase^(3)^. The methionine cycle plays a key role in the synthesis of amino acids methionine and cysteine as well as in the transfer of methyl groups to *S*-adenosylmethionine, a universal methyl donor for various methylation reactions, including DNA and histone methylation^(3)^. Therefore, these are referred to as 1C nutrients. In addition to folate, choline and methionine, 1C metabolism is mediated by vitamins B2, B6 and B12 (Fig. 1).

Inadequate intake of 1C nutrients is associated with metabolic perturbations at various stages of life. The consequences of folate deficiency during pregnancy on spontaneous abortion and neural tube defects have been well studied^(4)^. Choline inadequacy is associated with the risk of non-alcoholic fatty liver disease and age-related diseases, including Parkinson's and Alzheimer's diseases^(5,6)^. Inadequate intake of vitamins B2, B6 and B12 have been associated with anaemia and impaired angiogenesis^(7)^. Pregnant women with vitamin B12 deficiency were associated with having vitamin B12-deficient infants^(7,8)^.

Surveys of dietary intake of 1C nutrients have been concentrated in Western countries, whereas studies in Asia and Africa are limited, and results varied. Mean folate intake levels in countries without mandatory B vitamin fortification varied from 170 μg dietary folate equivalence (DFE)/d in Ethiopia to 246 and 499 μg DFE/d in Sweden and Korea, respectively^(9–11)^. Usual intake levels of vitamins B2, B6 and B12 were also adequate for most people in Korea^(12)^. In contrast, a national survey in Pakistan indicated that more than half of the population had folate and vitamin B12 deficiency^(13)^. The difference in 1C nutrient intake levels and status among countries could be due to the difference in caloric intake levels, access to fortified food and supplements as well as the difference in cut-off levels, dietary assessment methods and underlying food composition databases. In countries with mandatory B vitamin fortification such as South Africa and Canada, the programme was effective in reducing the prevalence of neural tube defects^(14,15)^. Although the intake of B vitamins in these countries are generally adequate, a small study indicated that 40 % of female college students and young women living in Vancouver, Canada had vitamin B2 deficiency^(16)^. Presently, Thailand does not have mandatory B vitamin fortification. A previous study in Bangkok, Thailand (*n* 182) indicated that median folate intake level was 128 μg DFE/d, which is below the Thai Estimated Average Requirement (EAR) of 220 μg DFE/d^(17,18)^. Another study also reported that the prevalence of folate and B12 deficiencies based on biochemical parameters were 38⋅8 and 0⋅6 %, respectively^(19)^.

Among the 1C nutrients, choline is a nutrient of concern for underconsumption^(20)^. Inadequate choline intake has been observed in the European and American populations. The average choline intake of European populations was well below the adequate intake (AI) level, and only 6⋅6 % of U.S. adults consumed choline more than the AI level^(21,22)^. Because choline is found in meat and meat products, Asian populations who generally consume less amounts of these food groups may also be at risk of choline inadequacy; however, studies surveying choline intake status are lacking in this part of the world.

The objective of the present study is to assess usual intake of 1C nutrients in young Thai adults. A cross-sectional survey was conducted on a sample of healthy Thai university students (*n* 246). Dietary, socioeconomic and anthropometric information was collected and used to assess the usual intake of 1C nutrients and their relationship with obesity measures.

## Materials and methods

### Study design and population

A cross-sectional descriptive study design was used to survey the usual intake levels of 1C nutrients in 246 healthy adults (82 male and 164 female) 19–30 years of age. Sample size was determined to differentiate mean choline intake levels between sex using data from our pilot study with the effect size of 60 mg/d, sd of 136⋅5 mg/d and at 0⋅05 α-level and 0⋅2 β-level. The inclusion criteria were students who were currently enrolled at Chiang Mai University, aged 19–30 years, had a Thai race and nationality, and had no chronic diseases. Exclusion criteria were individuals with habitual dietary restriction such as vegan, vegetarian, weight-loss, professional or university athletes, those who currently used medical prescriptions that may interfere with dietary behaviours, and pregnant or lactating women. Socioeconomic, health habit, anthropometric and dietary intake information was collected from all participants. Anthropometric and dietary data were assessed twice in a subgroup of participants (*n* 87; 34 men, 53 women) to estimate intra-individual variation and usual intake. All research personnel received training in human ethics research (Human Subject Protection Course). This study was conducted according to the guidelines laid down in the Declaration of Helsinki and all procedures involving human subjects/patients were approved by the Ethics Committee of the Faculty of Medicine, Chiang Mai University (Study Code: CHOLCMU/Research ID: NONE-2563-06999). Written informed consent was obtained from all subjects. The study was registered in the Thai Clinical Trials Registry (ID: TCTR20210420007).

### Study protocol

Participants were recruited from Chiang Mai University, Chiang Mai, Thailand via flyers and online postings. Study enrolments were regularly monitored for diversity of participants from various departments, and recruitment efforts were focused on under-represented groups, such as those who did not frequent the main campus area or post-graduate students. Screening was conducted using online questionnaires, and eligible participants were invited to a session at the Faculty of Agro-Industry at Chiang Mai University, Thailand.

During the session, the participants were asked to sign a consent form and provide socioeconomic information, health habits such as dietary patterns, physical activity levels, alcohol consumption, smoking status, screen time and sleep quality on a questionnaire. The participants then underwent anthropometric assessment by trained personnel. Weight and height were measured using a calibrated scale (Seca GmbH and Co. KG). Waist and hip circumferences were measured according to World Health Organization guidelines, that is, waist circumference (WC) was measured at the midpoint between the last palpable rib and iliac crest, and hip circumference was measured at the widest position of the bottom^(23)^. Finally, trained personnel administered a 24 h dietary recall interview. Food pictures, utensils and measuring cups were shown to participants to aid their recall process.

### Dietary intake assessment

Dietary intake information was assessed via 24 h dietary recall interviews. Study personnel collected information on all food items that the participants consumed during the past 24 h (midnight to midnight) using the validated Automated Self-Administered 24-Hour Dietary Assessment Tool (ASA24) developed by the National Cancer Institute, Bethesda, MD, USA. ASA24 utilised the Automated Multiple-Pass Method, in which a list of consumed food items was compiled, followed by time and occasion of eating, and detailed descriptions of each food, including preparation methods, portion sizes and addition^(24)^. A food-matching protocol was developed to ensure consistency.

### Usual intake of one-carbon nutrients

Dietary recall data were linked to the USDA food composition database, which contains data from the Food and Nutrient Database for Dietary Studies, Food Patterns Equivalents Database and NHANES Dietary Supplement Database^(25)^. To investigate the precision of food matching, the same set of dietary data was also linked to the Thai Food Composition Database via the INMUCAL-Nutrient program (version 4.0) developed by the Institute of Nutrition, Mahidol University, Thailand. Correlation coefficients (Pearson's *r*) between the Thai and U.S. databases were 0⋅62 (*P* < 0⋅0001) for energy, 0⋅58 (*P* < 0⋅0001) for carbohydrate, 0⋅82 (*P* < 0⋅0001) for protein, 0⋅50 (*P* < 0⋅0001) for fat, 0⋅47 (*P* < 0⋅0001) for calcium, 0⋅54 (*P* < 0⋅0001) for phosphorus, 0⋅32 (*P* = 0⋅002) for iron, 0⋅57 (*P* < 0⋅0001) for potassium, 0⋅63 (*P* < 0⋅0001) for sodium, 0⋅50 (*P* < 0⋅0001) for copper, 0⋅16 (*P* = 0⋅13) for magnesium, 0⋅14 (*P* = 0⋅19) for selenium, 0⋅51 (*P* < 0⋅0001) for zinc, 0⋅41 (*P* < 0⋅0001) for niacin, 0⋅35 (*P* = 0⋅001) for vitamin B1, 0⋅56 (*P* < 0⋅0001) for vitamin B2, 0⋅31 (*P* = 0⋅003) for vitamin B6, and 0⋅33 (*P* = 0⋅002) for vitamin B12.

The ASA24 nutrient intake data were used to calculate the usual intake of each 1C nutrient following the Multiple Source Method (MSM). MSM is a method for estimating the usual dietary intake from a 24 h recall or short-term assessments. This method can account for intra-individual variation and handle different distributions of nutrient data^(26)^. Moreover, usual intake can be modelled using repeated measurements or long-term assessments at the individual level^(26)^.

### Statistical analyses

Dietary data were inspected for outliers and extreme values. Health habit information was either categorised or used as a continuous variable. Specifically, smoking status was categorised into current or non-smokers based on smoking activity in the past 12 months. Vigorous physical activity was defined as an activity that exerts force and greatly increases respiration or heart rates, such as lifting or carrying heavy objects, building, plowing, running, playing basketball, for at least 10 min continuously. Moderate physical activity was defined as an activity that slightly increases respiration or heart rates, such as carrying small objects, fast-walking, cycling, swimming, golfing, for at least 10 min continuously. These definitions were clearly written in the questionnaires, and the obtained data was converted to hour/week. Alcohol consumption and screen time responses were numerical and based on the past 12 months and the past 30 d, respectively.

Descriptive statistics (means, sds, frequency, counts and histograms) were generated for the whole dataset and for subgroups of interest. Differences between group means were analysed using *t*-test or ANOVA. The χ^2^ test was used for categorical variables. Associations between each 1C nutrient intake and obesity measures (body mass index (BMI), WC and waist-to-hip ratio (WHR)) were analysed using multiple linear regression models adjusted for age, sex and energy intake. Because energy intake was included as a covariate, nutrient variables entered into the model were not pre-adjusted for energy. Correlations between the U.S. and Thai databases were analysed using Pearson's correlation method. All analyses were conducted using SPSS (version 17) at a significance threshold of *P* < 0⋅05.

## Results

### Participants socioeconomic status, health characteristics and dietary intake

The study participants had a mean age of 21 years, with most being undergraduate students who had not worked part-time and were living alone (Table 1). Most participants were female, had family income (i.e. income of their parents) between 20 000 (~580 USD) and 49 999 baht (~1450 USD) per month, and had an average personal monthly income or stipend of 6858 ± 4602 (mean ± sd) baht (200 ± 134 USD) per month. Male and female participants did not differ in any measured socioeconomic variables (*P* > 0⋅08), except housing, in which male participants were more likely to live with roommates, while females were more likely to live alone (*P* < 0⋅05).

**Table 1.** Participant characteristics, health patterns and dietary intake (*n* 246) by sex^†^

	Male (*n* 82)	Female (*n* 164)	Total (*n* 246)	*P*-value
*Demographics and socioeconomics*				
Age (years)	21⋅6 ± 1⋅6	21⋅2 ± 1⋅4	21⋅4 ± 1⋅5	0⋅07
Education (%, *n*)				
Undergraduate level	87⋅8, 72	94⋅5, 155	92⋅3, 227	0⋅06
Graduate level	12⋅2, 10	5⋅50, 9	7⋅70, 19	
Having part-time jobs (%, *n*)	14⋅6, 12	12⋅8, 21	13⋅4, 33	0⋅69
Number of family members	4⋅22 ± 1⋅0	4⋅14 ± 1⋅1	4⋅17 ± 1⋅1	0⋅60
Monthly family income (%, *n*)				
<20 000 baht (~580 USD)	11⋅0, 9	21⋅3, 35	17⋅9, 44	0⋅08
20 000–49 999 baht (580–1450 USD)	45⋅1, 37	39⋅0, 64	41⋅1, 101	
50 000–79 999 baht (1,450–2320 USD)	31⋅7, 26	28⋅7, 47	29⋅7, 73	
>80 000 baht (~2320 USD)	12⋅2, 10	11⋅0, 18	11⋅4, 28	
Monthly income or stipend (baht)	7007 ± 4815	6784 ± 4507	6858 ± 4602	0⋅72
Housing (%, *n*)^‡^				
Living with family	20⋅7, 17	20⋅7, 34	20⋅7, 51	0⋅60
Living with roommate(s)	40⋅2, 33	37⋅2, 61	38⋅2, 94	
Living alone	32⋅9, 27	41⋅5, 68	38⋅6, 95	
*Health status and behaviours*				
Smoking status (%, *n*)				
Non-smoker	86⋅4, 70	95⋅1, 155	92⋅2, 225	0⋅02
Current smoker	13⋅6, 11	4⋅90, 8	7⋅80, 19	
Alcohol consumption (drink/week)	12⋅2 ± 46	12⋅0 ± 58	12⋅1 ± 54	0⋅98
Vigorous physical activity (hour/week)	2⋅47 ± 3⋅1	0⋅721 ± 1⋅3	1⋅31 ± 2⋅2	<0⋅0001
Moderate physical activity (hour/week)	1⋅78 ± 2⋅9	1⋅44 ± 2⋅0	1⋅55 ± 2⋅4	0⋅29
Screen time (hour/week)	9⋅13 ± 4⋅7	10⋅8 ± 4⋅4	10⋅3 ± 4⋅6	0⋅01
BMI (kg/m^2^)	22⋅2 ± 5⋅2	21⋅8 ± 4⋅6	21⋅9 ± 4⋅8	0⋅47
BMI categories (%, *n*)				
Underweight (<18⋅5 kg/m^2^)	11⋅0, 9	22⋅6, 37	18⋅7, 46	0⋅09
Normal (18⋅5–22⋅9 kg/m^2^)	46⋅3, 38	48⋅2, 79	47⋅6, 117	
Overweight (23⋅0–24⋅9 kg/m^2^)	25⋅6, 21	14⋅6, 24	18⋅3, 45	
Obese (25⋅0–29⋅9 kg/m^2^)	12⋅2, 10	9⋅10, 15	10⋅2, 25	
Severely obese (≥30⋅0 kg/m^2^)	4⋅90, 4	5⋅50, 9	5⋅30, 13	
Waist circumference (cm)	32⋅5 ± 4⋅5	29⋅3 ± 4⋅6	30⋅4 ± 4⋅8	<0⋅0001
Waist-to-hip ratio	0⋅83 ± 0⋅06	0⋅77 ± 0⋅05	0⋅79 ± 0⋅06	<0⋅0001
Risk of visceral adiposity (%, *n*)^§^				
Low	95⋅1, 78	76⋅2, 125	82⋅5, 203	0⋅0002
Moderate–high	4⋅90, 4	23⋅8, 39	17⋅5, 43	
*Intake per day*				
Energy (kcal)	1850 ± 711	1548 ± 670	1648 ± 697	0⋅001
Carbohydrate (g)	216 ± 74	195 ± 92	202 ± 87	0⋅08
Protein (g)	88⋅3 ± 41	70⋅3 ± 31	76⋅3 ± 36	0⋅0002
Total fat (g)	67⋅5 ± 35	54⋅5 ± 31	58⋅8 ± 33	0⋅003
Sodium (mg)	4097 ± 1631	3403 ± 1674	3634 ± 1688	0⋅002
Vitamin B1 (mg)	1⋅79 ± 0⋅97	2⋅33 ± 8⋅7	2⋅15 ± 7⋅1	0⋅58
Vitamin B2 (mg)	1⋅85 ± 0⋅95	2⋅42 ± 8⋅7	2⋅23 ± 7⋅1	0⋅55
Vitamin B6 (mg)	1⋅96 ± 1⋅0	2⋅49 ± 8⋅7	2⋅31 ± 7⋅1	0⋅58
Vitamin B12 (μg)	6⋅20 ± 9⋅5	6⋅28 ± 12	6⋅26 ± 12	0⋅96
Choline (mg)	418 ± 191	337 ± 164	364 ± 178	0⋅001
Folate (μg dietary folate equivalent)	195 ± 154	155 ± 101	168 ± 122	0⋅02

† Values are mean ± sds for continuous variables and % and *n* for categorical variables. *T*-test and χ^2^ test were used to evaluate group differences for the respective groups, with a significance threshold of *P* < 0⋅05.

‡ Missing values are *n* 5 for males and *n* 1 for females.

§ Visceral adiposity risk categories were based on wait-to-hip ratios as follows: low (female ≤0⋅80; male ≤0⋅95), moderate (female 0⋅81–0⋅85; male 0⋅96–1⋅00) and high (female >0⋅85; male >1⋅00).

The participants were mostly non-smokers and had a mean alcohol consumption of 12 drinks/week. Males were more likely to be current smokers than females (*P* = 0⋅02). Participants reported mean vigorous and moderate physical activities of 1⋅31 ± 2⋅2 and 1⋅55 ± 2⋅4 h/week, respectively. Male participants reported more vigorous physical activity (*P* < 0⋅0001) and shorter screen times (*P* = 0⋅01) than female participants. Mean BMI, WC and WHR lied within normal ranges (21⋅9 ± 4⋅8 kg/m^2^, 30⋅4 ± 4⋅8 cm and 0⋅79 ± 0⋅06, respectively). However, 23⋅8 % of female participants had a moderate to high risk of visceral adiposity compared to 4⋅9 % of male participants (*P* = 0⋅0002). Regarding dietary intake, male participants consumed more energy, protein, fat, sodium, folate and choline than female participants (*P* ≤ 0⋅008).

### Usual intake and prevalence of inadequacy of one-carbon nutrients

**Figure 2.**
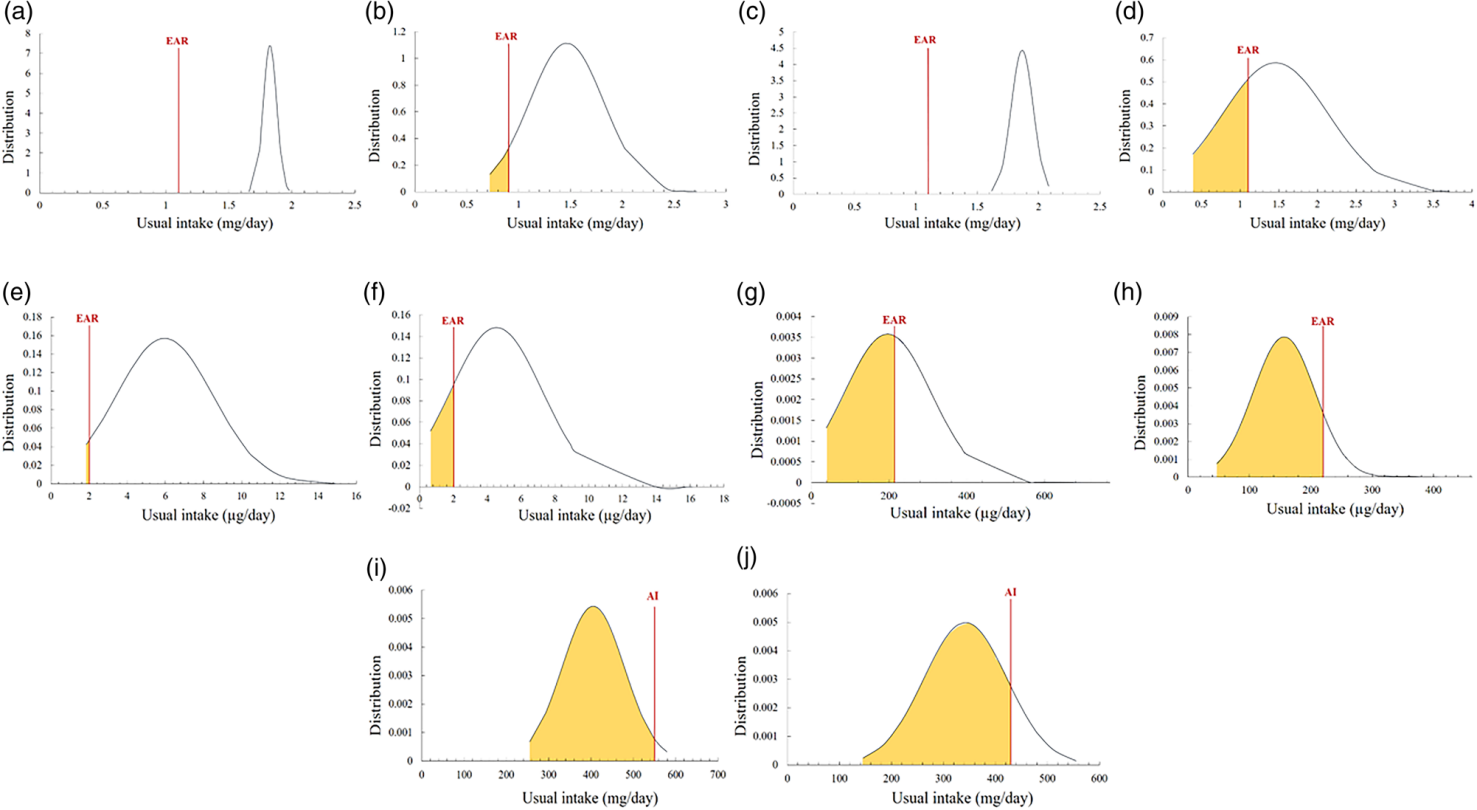
Usual intake distribution of one-carbon nutrients by sex: vitamins B2 (a–male, b–female), B6 (c–male, d–female) and B12 (e–male, f–female), dietary folate (g–male, h–female) and choline (i–male, j–female). Lines indicated the estimated average requirement (EAR; for vitamins B2, B6 and B12, folate) or the adequate intake (AI; for choline) based on the U.S. National Academy of Medicine. Yellow areas indicated a proportion of samples that had inadequate intake of each nutrient.

Mean and median usual intakes of vitamins B2, B6 and B12 among participants were higher than the EAR set by the U.S. National Academy of Medicine, which is also used for Thai Dietary Reference Intake (Fig. 2; Tables 2 and 3). The prevalence of vitamins B2, B6 and B12 inadequacy, calculated using the EAR cut-point method, ranged from 8⋅54 to 31⋅7 %, indicating that most of the participants consumed adequate amounts of these vitamins. However, an inadequate intake of folate and potentially choline has been observed. The mean usual intake of folate was 195 and 155 μg DFE/d among male and female participants, respectively. When compared with the Thai EAR of 220 μg DFE/d, these results indicate that 74⋅4 and 81⋅1 % of men and women did not consume adequate amounts of folate, respectively. Unlike other 1C nutrients, choline EAR has not yet been established; therefore, the prevalence of inadequacy cannot be determined. Nonetheless, current observations indicate that 79⋅3 % of men and 76⋅2 % of women do not meet their respective AI levels and are potentially at risk of inadequacy.

**Table 2.** Usual one-carbon nutrient intake by characteristic and health subgroups^†^

	Vitamin B2 (mg/d)		Vitamin B6 (mg/d)		Vitamin B12 (μg/d)		Folate (μg dietary folate equivalent/d)		Choline (mg/d)	
	Mean^†^±sd	*P*-value	Mean^†^±sd	*P*-value	Mean^†^±sd	*P*-value	Mean^†^±sd	*P*-value	Mean^†^±sd	*P*-value
Sex										
Male	1⋅85 ± 0⋅95	0⋅55	1⋅96 ± 1⋅0	0⋅58	6⋅20 ± 9⋅5	0⋅96	195^a^ ± 154	0⋅02	418 ± 191	0⋅001
Female	2⋅42 ± 8⋅7		2⋅49 ± 8⋅7		6⋅28 ± 12		155^b^ ± 101		337 ± 164	
Education										
Undergraduate level	2⋅31 ± 7⋅4	0⋅57	2⋅36 ± 7⋅4	0⋅70	11⋅9 ± 0⋅79	0⋅22	164 ± 107	0⋅32	368 ± 176	0⋅24
Graduate level	1⋅35 ± 0⋅70		1⋅71 ± 1⋅5		3⋅20 ± 0⋅73		220 ± 238		317 ± 195	
Having part-time jobs										
Yes	1⋅63 ± 0⋅77	0⋅60	1⋅84 ± 1⋅3	0⋅68	3⋅95 ± 3⋅1	0⋅22	183 ± 164	0⋅47	359 ± 186	0⋅86
No	2⋅32 ± 7⋅6		2⋅39 ± 7⋅6		6⋅61 ± 12		166 ± 115		365 ± 177	
Monthly family income (baht)										
<20 000 baht (~580 USD)	1⋅46 ± 0⋅64		1⋅63 ± 0⋅80		4⋅37 ± 4⋅1		140 ± 80		316 ± 140	
20 000–49 999 baht (580–1450 USD)	2⋅68 ± 9⋅9	0⋅54	2⋅69 ± 9⋅9	0⋅67	7⋅35 ± 14	0⋅20	163 ± 92	0⋅20	372 ± 166	0⋅12
50 000–79 999 baht (1,450–2320 USD)	1⋅64 ± 1⋅0		1⋅83 ± 1⋅2		4⋅87 ± 5⋅3		184 ± 160		392 ± 213	
>80 000 baht (~2320 USD)	3⋅38 ± 9⋅3		3⋅29 ± 9⋅4		8⋅88 ± 18		192 ± 152		339 ± 158	
Housing										
Living with family	1⋅53 ± 0⋅79		1⋅47 ± 0⋅60		6⋅66 ± 14		164 ± 121		365 ± 176	
Living with roommate(s)	3⋅10 ± 11	0⋅51	3⋅26 ± 11	0⋅43	6⋅37 ± 12	0⋅98	163 ± 126	0⋅85	340 ± 156	0⋅36
Living alone	1⋅78 ± 1⋅0		1⋅85 ± 1⋅0		6⋅00 ± 9⋅8		175 ± 120		386 ± 200	
Smoking status										
Non-smoker	2⋅28 ± 7⋅4	0⋅73	2⋅31 ± 7⋅4	1⋅00	6⋅37 ± 12	0⋅66	162 ± 105	0⋅17	356 ± 173	0⋅03
Current smoker	1⋅70 ± 0⋅75		2⋅29 ± 1⋅5		5⋅17 ± 4⋅7		241 ± 241		450 ± 216	
BMI categories										
Underweight (<18⋅5 kg/m^2^)	2⋅74 ± 7⋅3		2⋅63 ± 7⋅4		6⋅16 ± 11		144 ± 64		357 ± 188	
Normal (18⋅5–22⋅9 kg/m^2^)	2⋅46 ± 9⋅2		2⋅57 ± 9⋅2		6⋅68 ± 14		166 ± 112		355 ± 161	
Overweight (23⋅0–24⋅9 kg/m^2^)	1⋅63 ± 0⋅87	0⋅92	1⋅88 ± 1⋅2	0⋅95	5⋅77 ± 8⋅8	0⋅98	193 ± 181	0⋅44	407 ± 233	0⋅48
Obese (25⋅0–29⋅9 kg/m^2^)	1⋅66 ± 0⋅80		1⋅68 ± 0⋅67		5⋅30 ± 5⋅7		176 ± 123		357 ± 133	
Severely obese (≥30⋅0 kg/m^2^)	1⋅56 ± 0⋅94		1⋅65 ± 0⋅84		6⋅30 ± 6⋅1		174 ± 116		330 ± 139	
Risk of visceral adiposity^‡^										
Low	2⋅39 ± 7⋅8	0⋅45	2⋅46 ± 7⋅8	0⋅49	6⋅34 ± 12	0⋅81	169 ± 120	0⋅83	368 ± 176	0⋅38
Moderate–high	1⋅48 ± 0⋅92		1⋅63 ± 0⋅83		5⋅88 ± 12		165 ± 131		342 ± 183	

† Values are the mean ± sds for continuous variables. Analysis of Variance was used to evaluate differences between subgroups with a significance threshold of *P* < 0⋅05, and superscript letters denote differences between levels.

‡ Visceral adiposity risk categories were based on wait-to-hip ratios as follows: low (female ≤0⋅80; male ≤0⋅95), moderate (female 0⋅81–0⋅85; male 0⋅96–1⋅00) and high (female >0⋅85; male >1⋅00).

**Table 3.** Median, 5th percentile and 95th percentile of usual one-carbon nutrient intake and percentage of participants with inadequate intake

One-carbon nutrients		Median ± sd	Usual one-carbon nutrient intake percentiles		%, *n* of participants with intake below EAR^†^
			5th	95th	
Vitamin B2 (mg/d)	Male	1⋅9 ± 0⋅7	1⋅1	2⋅8	14⋅6, 12
	Female	2⋅1 ± 3⋅7	0⋅8	3⋅3	18⋅3, 30
Vitamin B6 (mg/d)	Male	2⋅0 ± 0⋅4	1⋅4	2⋅7	8⋅54, 7
	Female	2⋅2 ± 5⋅0	0⋅8	3⋅5	31⋅7, 52
Vitamin B12 (μg/d)	Male	6⋅3 ± 4⋅5	2⋅1	12	19⋅5, 16
	Female	6⋅0 ± 5⋅6	2⋅1	13	31⋅1, 51
Folate (μg dietary folate equivalent/d)	Male	197 ± 111	96	387	74⋅4, 61
	Female	156 ± 51	95	241	81⋅1, 133
Choline (mg/d)	Male	420 ± 132	232	655	79⋅3, 65
	Female	339 ± 103	191	513	76⋅2, 125

† Adequate intake was used for choline.

Men consumed more folate and choline than women (*P* ≤ 0⋅01; Table 2). Although most 1C nutrient intakes did not differ by socioeconomic and health characteristics, the choline intake level of current smokers was higher than that of non-smokers (*P* = 0⋅03).

### Relationships between one-carbon nutrient intake and obesity measures

Usual 1C intake levels were not associated with BMI, WC or WHR in the multiple linear regression models adjusted for sex, education, smoking, family income, age and energy intake (Table 4). However, a modification effect by sex was observed for vitamin B2 (*P*-interaction = 0⋅002) and choline (*P*-interaction = 0⋅02), where every 1-mg increase in vitamin B2 and 100-mg increase in choline intake were associated with 2⋅07 (*P* = 0⋅01) and 0⋅81 kg/m^2^ (*P* = 0⋅04), respectively, decrease in BMI in male participants (Table 5). Vitamins B6, B12 and folate intakes were not associated with BMI, WC and WHR in either sex.

**Table 4.** Associations between usual intake levels of one-carbon nutrients and body mass index, waist circumference and waist-to-hip ratios^†^

	BMI		Waist circumference		Waist-to-hip ratio	
	Beta	*P*-value^†^	Beta	*P*-value^†^	Beta	*P*-value
Vitamin B2 (mg/d)	−0⋅03	0⋅57	−0⋅005	0⋅90	0⋅0003	0⋅48
Vitamin B6 (mg/d)	−0⋅02	0⋅60	−0⋅006	0⋅89	0⋅0002	0⋅60
Vitamin B12 (μg/d)	0⋅0003	0⋅99	−0⋅008	0⋅75	<0⋅0001	0⋅89
Choline (100 mg/d)	−0⋅29	0⋅19	−0⋅19	0⋅36	0⋅001	0⋅56
Folate (100 μg dietary folate equivalent/d)	0⋅19	0⋅55	0⋅03	0⋅92	0⋅004	0⋅26

† Linear regression models adjusted for age, sex, education, smoking, family income and energy intake were used, with a significance threshold of *P* < 0⋅2.

**Table 5.** Associations between usual intake levels of one-carbon nutrients and body mass index, waist circumference and waist-to-hip ratios (WHR) by sex^†^

1C Nutrient	BMI		Interaction (1C nutrient * sex)	Waist circumference		Interaction (1C nutrient * sex)	WHR		Interaction (1C nutrient * sex)
	Beta	*P*-value	*P*-value	Beta	*P*-value	*P*-value	Beta	*P*-value	*P*-value
Vitamin B2 (mg/d)									
Male	−2⋅07	0⋅01	0⋅002	−0⋅45	0⋅53	0⋅37	−0⋅005	0⋅62	0⋅88
Female	−0⋅023	0⋅59		−0⋅009	0⋅84		−0⋅0003	0⋅42	
Vitamin B6 (mg/d)									
Male	−0⋅64	0⋅37	0⋅14	0⋅39	0⋅53	0⋅87	0⋅007	0⋅38	0⋅18
Female	−0⋅025	0⋅56		−0⋅012	0⋅77		−0⋅0003	0⋅47	
Vitamin B12 (μg/d)									
Male	0⋅010	0⋅88	0⋅86	−0⋅016	0⋅78	0⋅89	0⋅0003	0⋅74	0⋅49
Female	−0⋅004	0⋅88		−0⋅012	0⋅68		<0⋅0001	0⋅84	
Choline (100 mg/d)									
Male	−0⋅81	0⋅04	0⋅02	0⋅098	0⋅78	0⋅85	−0⋅0002	0⋅96	0⋅80
Female	0⋅02	0⋅94		−0⋅36	0⋅16		0⋅002	0⋅59	
Folate (100 μg dietary folate equivalent/d)									
Male	0⋅32	0⋅50	0⋅74	0⋅23	0⋅57	0⋅80	0⋅006	0⋅26	0⋅44
Female	0⋅03	0⋅94		−0⋅21	0⋅62		0⋅002	0⋅68	

† Linear regression models adjusted for age, sex, education, smoking, family income and energy intake were used with a significant threshold of *P* < 0⋅05.

## Discussion

### Folate intake

The findings in the present study revealed that the majority of participants had inadequate folate intake. This is consistent with a previous study in Thai women of childbearing age, which reported that 65⋅5 % of women had low dietary folate levels and 18 % had low serum folate^(27)^. Since dietary folate has been correlated with serum folate levels in a Thai population^(27)^, our results imply that a significant proportion of female participants may have had low folate status and may be at a greater risk of deficiency than populations with a higher intake level. The inadequate folate intake observed in this study was due to dietary patterns that were low in fruits and vegetables. Intake of these food groups has been shown to be positively associated with folate status^(10)^. The study participants often reported consuming white rice, white meat, snacks and desserts, while sources of folate, such as cereals, whole grains, dark green vegetables and fish, were not frequently consumed. Male participants consumed more folate (and calories) than females and thus were less likely to be inadequate. Lower folate intake was also observed in Western populations. In Sweden, the median folate intake was 246 μg DFE/d, which was adequate when compared to its EAR of 200 μg DFE/d^(10)^, this cut-off is lower than that of several countries.

### Choline intake

To our knowledge, this is the first study to estimate choline intake in a Thai population. Our results indicated that the mean choline intake was well below the AI levels for both men and women. This was likely due to the low consumption of choline food sources and not due to inadequate caloric intake, since most participants consumed enough energy according to the Thai Recommended Daily Intake. Indeed, the participants reported consuming small amounts of choline food such as eggs, beef, liver and other organ meat. This observation was consistent with a previous study that found that most Thai people consumed eggs only 3–4 day per week, or approximately ½−1 egg per day^(28)^.

Underconsumption of choline was also observed in Western populations, with generally higher caloric and protein intake than in Thai populations. The mean usual choline intake in U.S. adults aged 19–30 years was 392 mg/d for men and 257 mg/d for women, and people who consumed eggs had almost twice as much choline intake as non-consumers^(22)^. Additionally, an analysis of 12 surveys collected in Europe indicated that adult men aged 18–65 years had an average choline intake of 357–468 mg/d, whereas adult women had 291–374 mg/d of choline^(21)^. Similarly, the mean choline intake in the Australian population was 310 and 248 mg/d for 19–64-year-old males and females, respectively^(29)^. These studies collectively indicate that choline intake is suboptimal in populations worldwide. Choline deficiency is associated with the risk of non-alcoholic fatty liver disease, nervous system problems and abnormal 1C metabolism^(30,31)^, and higher choline intake has been associated with improved inflammatory marker concentrations in healthy adults and 1C metabolites in pregnant women^(32,33)^. In our study, a negative association between choline intake and BMI was observed in male participants. This is consistent with results from a recent study in Poland where choline intake was associated with lower body fat mass and WC as well as sex-dependent associations between 1C nutrient intake and status with anthropometric indicators^(34)^. Our study population contributed evidence showing similar trends were observed across different geographical and racial groups, suggesting that these relationships have underlying biological origins.

### Intake of other B vitamins

The intake of vitamins B2, B6 and B12 was adequate among study participants and was higher in men than in women. This is expected, as men also consume more calories than women. The intake of these B vitamins was not associated with socioeconomic backgrounds and health behaviours, suggesting that the overall dietary quality was homogenous in this sample. The lack of associations between vitamin B intake and obesity measures (BMI, WC and WHR) may be due to the small sample size and sample homogeneity. Nonetheless, an inverse association between vitamin B2 intake and BMI was observed in male participants. Vitamin B2 is a coenzyme of methylenetetrahydrofolate reductase (MTHFR), the rate-limiting enzyme in the folate cycle (Fig. 1). Evidence in *mthfr* heterozygous knockout mice showed that males had a greater turnover of choline and increased its use as a methyl group donor when dietary folate was restricted. It is possible that male participants who had adequate folate intake and consumed higher B2 levels had optimal MTHFR activity, thus sparing choline for use in other pathways that support fat metabolism. This hypothesis also explains the beneficial association between choline intake and BMI in males^(35)^.

## Conclusion

Usual intake of 1C nutrients in male and female young Thai adults was adequate for vitamins B2, B6 and B12. The majority of participants had inadequate folate intake and did not achieve recommended intake levels for choline. The intake levels of vitamin B2 and choline were inversely associated with BMI in men. These findings are in line with reports in Western countries, suggesting that inadequate intake of folate and choline is not geographically constrained.
